# Supplementary material for: Identification of Freezing-Responsive microRNAs and Their Targets in Chinese Jujube by Small RNA and Degradome Sequencing
Source: Int J Mol Sci. 2026 Sep 4;27(17):7912. doi: 10.3390/ijms27177912 (PMC13566890; doi:10.3390/ijms27177912)
Supplement: Supplementary file 1 [file ijms-27-07912-s001.zip › Supplementary Material S2/novel_miR_209.pdf]

Provisional ID	:	novel_miR_209
Score total	:	24.1
Score for star read(s)	:	3.9
Score for read counts	:	21.5
Score for mfe	:	0.9
Score for randfold	:	-2.2
Score for cons. seed	:	
Total read count	:	54
Mature read count	:	50
Loop read count	:	3
Star read count	:	1

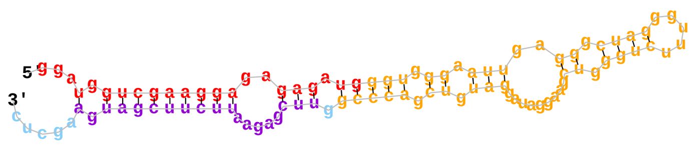

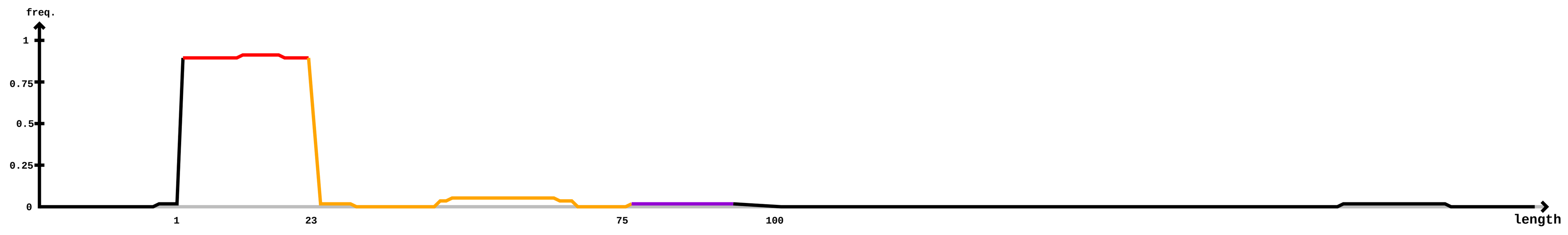

Mature Star

```
5'- caugagcuugguggguggguaaggauggucgaaggagagagaugggugggaauugagggcuaggguuucugggucgaaggauaggaugucgacccgguucgagaauucuucgaugaagcucccgacggcgauucggugguggcgcguucgugccggugaggaggaggacaaggccauggcguauaaaggauaaggggauucauaagucaccgaauuaucggaucugagaugagauuccugcgucgaauuu  -3'  obs
    caugagcuugguggguggguaaggauggucgaaggagagagaugggugggaauugagggcuaggguuucugggucgaaggauaggaugucgacccgguucgagaauucuucgaugaagcucccgacggcgauucggugguggcgcguucgugccggugaggaggaggacaaggccauggcguauaaaggauaaggggauucauaagucaccgaauuaucggaucugagaugagauuccugcgucgaauuu        exp
    ..((((.(((.((..((((..((..(.(((((((((..(((.(((((.((.(((..((.((((.....)))).))........))).)).))))).))).....)))))))).)..)))))).)).)))))))((.(((((..((((...((.....))....))))...))))).))........(((..((((((((((...(((((((....))))...))).)))).))))))..)))......   reads  mm  sample
    ..................guaaggauggucgaaggagag...........................................................................................................................................................................................................   1      0   LT-1
    ......................ggauggucgaaggagagagaug..............................................................................................................................................................................................   25     0   LCK-2
    ......................ggauggucgaaggagagagaug..............................................................................................................................................................................................   25     0   LCK-1
    ................................aggaAagagaugggugggа.......................................................................................................................................................................................   1      1   JT-3
    .................................................................uuucugggucgaaggauaggaug..................................................................................................................................................................   2      0   LT-2
    ...................................................................ucugggucgaaggauagg.....................................................................................................................................................................   1      0   LT-3
    .................................................................................................uucgagaauucuucAauga......................................................................................................................................   1      1   JT-1
    ........................................................................................................................................................................................................................aucggaucugagaugaga................   1      0   LT-1
```
